# Identification of ABCA5 among ATP-Binding Cassette Transporter Family as a New Biomarker for Colorectal Cancer

**DOI:** 10.1155/2022/3399311

**Published:** 2022-06-22

**Authors:** Peilong Bu, Yafei Xiao, Shaowen Hu, Xiaowei Jiang, Cong Tan, Mengyuan Qiu, Wanting Huang, Mengmeng Li, Quanying Li, Changjiang Qin

**Affiliations:** ^1^Department of General Surgery, Huaihe Hospital of Henan University, Kaifeng 475000, China; ^2^Department of Pediatric Orthopedics, The Third Affiliated Hospital of Zhengzhou University, Zhengzhou 450000, China; ^3^Institute of Biomedical Informatics, Cell Signal Transduction Laboratory, Bioinformatics Center, Henan Provincial Engineering Center for Tumor Molecular Medicine, School of Basic Medical Sciences, Henan University, Kaifeng 475004, China

## Abstract

**Background:**

The increasing incidence and mortality of colorectal cancer (CRC) urgently requires updated biomarkers. The ABC transporter family is a widespread family of membrane-bound proteins involved in the transportation of substrates associated with ATP hydrolysis, including metabolites, amino acids, peptides and proteins, sterols and lipids, organic and inorganic ions, sugars, metals, and drugs. They play an important role in the maintenance of homeostasis in the body.

**Purpose:**

This study aims to search for new markers in the ABC transporter gene family for diagnostic and prognostic purposes through data mining of The Cancer Genome Atlas (TCGA) and GEO (Gene Expression Omnibus) datasets.

**Methods:**

A total of 980 samples, including 684 CRC patients and 296 controls from five different datasets, were included for analysis. The construction of the PPI (protein-protein interaction) network and pathway analysis were performed in STRING database and DAVID (database for annotation, visualization, and integrated discovery), respectively. In addition, GSEA (gene set enrichment analysis) and WGCNA (weighted gene co-expression network analysis) were also used for functional analysis.

**Results:**

After several rounds of screening and validation, only the *ABCB5* gene was retained among the 49 genes.

**Conclusions:**

The results demonstrated that *ABCA5* expression is reduced in CRC and patients with high *ABCA5* expression have better OS, which can provide guidance for better management and treatment of CRC in the future.

## 1. Introduction

Colorectal cancer (CRC), the third most commonly diagnosed cancer and the second leading cause of cancer death worldwide, was diagnosed in 1931,590 cases and 935,173 people died of colorectal cancer worldwide in 2021 [1]. Due to the early development of metastasis, the overall survival time of patients with CRC is generally less than 5 years [2]. Although CRC can be treated by surgery, radiotherapy, chemotherapy, immunotherapy, and other comprehensive treatment methods, the drug resistance of tumor and a series of side effects often offset the curative efficacy of the treatment schemes, resulting in a high recurrence rate (54.5%) [3] and high mortality rate (9.5%) [4]. Due to the lack of precise and effective molecular targets for CRC treatment, it remains important to explore new diagnostic, prognostic biomarkers.

The ABC transporter family is a kind of widespread membrane-bound protein, mainly distributed in the liver, intestines, blood-brain barrier, blood testosterone barrier, placenta, and kidney [5], and participates in the transportation of ATP hydrolysis-related substrates [6]. The energy obtained from ATP hydrolysis by ABC is used to overcome the concentration gradient and transport the substrates across the outer and inner membrane [7]. ABC proteins promote the transportation of heterogeneous substrates across cell membranes, including metabolites, amino acids, peptides and proteins, sterols and lipids, organic and inorganic ions, sugars, metals, and drugs [8]. The mechanism of ABC transporters plays an irreplaceable part in the formation of multidrug resistance [9].

ABC family genes are often used as a search for biomarkers due to their important biological functions. In the digestive system, Guo et al. found that *ABCA8* could be used as a prognostic biomarker for gastric adenocarcinoma and was associated with immune invasion [10]. In the urinary system, our group has previously identified *ABCG1* as a diagnostic and prognostic marker for clear cell renal cell carcinoma [11]. In this study, we are focusing on the expression of ABC genes in several datasets to evaluate their diagnostic and prognostic value in CRC.

## 2. Material and Methods

**Figure 1:**
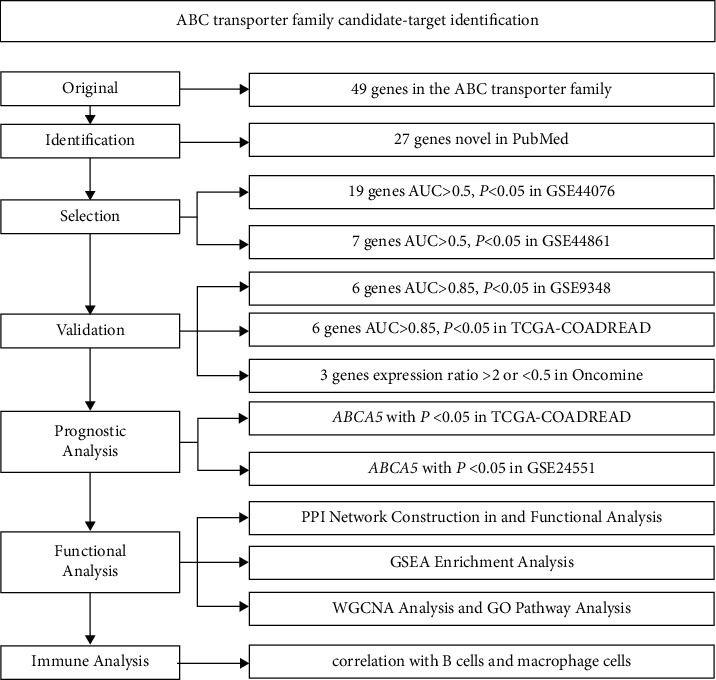
Procedure for the selection of the potential biomarker in CRC.

A total of 980 samples, including 684 CRC patients and 296 controls from five different datasets (GSE44076, GSE44861, GSE9348, TCGA-COADREAD, and GSE24551), were included for analysis. The further verification of mRNA level for the ABC gene was conducted on the Oncomine database (https://www.oncomine.org). Next, the prognostic analysis was performed in the TCGA Colon and Rectal Cancer (TCGA-COADREAD) dataset from UCSC Xena (https://xenabrowser.net). The construction of the PPI network and pathway analysis were performed in the STRING database and DAVID, respectively. In addition, gene set enrichment analysis (GSEA) and weighted correlation network analysis (WGCNA) were also used for functional analysis. Finally, immuno-infiltration analysis was conducted in the TIMER database (https://cistrome.shinyapps.io/timer/). The *t*-test is a simple, statistically based method for detecting differentially expressed genes [12]. The ROC curve is an analytical method, represented graphically, for assessing the overall diagnostic performance of a test and comparing the performance of two or more diagnostic tests [13]. Our screening conditions are progressively more lenient to more stringent, so the AUC threshold varies from 0.5 to 0.85, which naturally leads to a gradual reduction in the number of eligible genes. This serves two purposes: (1) to leave markers with high sensitivity and specificity; and (2) to reduce marker misses due to differences in the datasets. Different datasets, due to the existence of different instruments for the experiments, different proficiency of the experimenters, and even different reagents, can result in heterogeneity between different datasets (Figure S1). In addition, the validation steps are sequential, and some genes that fail to meet the conditions are eliminated, with those that meet all the conditions of the validation steps being retained in the end. A flow diagram for this study is shown in Figure 1.

### 2.1. Identification of Genes That Have Not Been Reported in CRC

Search for ABC family genes associated with CRC in PubMed (https://pubmed.ncbi.nlm.nih.gov/) on June 20, 2021. Our search strategy was as follows: (“colorectal cancer” OR “colon cancer” OR “colorectal carcinoma” OR “colorectal neoplasms” OR “colonic neoplasms” OR “rectal neoplasms” OR “CRC” OR “colon tumor” OR “rectal tumor”) AND (XXX), with XXX representing the specific ABC family gene. The ABC family genes that were not found to be markers for the diagnosis or prognosis of colorectal cancer were defined as novel genes. Then, these novel genes would be screened and identified as follows.

### 2.2. Screening of ABC Genes in the GEO Database

ROC analysis and *t*-test were conducted in the GSE44076 dataset (98 patients with CRC and 98 controls) and the GSE44861 dataset (56 CRC patients and 56 controls), respectively. In the ROC analysis, genes with area under the curve (AUC) > 0.50 and *t*-test ^*∗∗*^*P* < 0.01 were deemed statistically significant and will undergo the next round of verification.

### 2.3. Three Rounds of Validation

The first round of verification was to perform *t*-test and ROC analysis in the GSE9348 dataset (70 CRC patients and 12 control groups). Genes with *P* < 0.05 and AUC > 0.85 were considered to have statistical significance. The second round of validation was conducted in the TCGA-COADREAD dataset (380 patients with CRC and 51 controls). Genes with ^*∗∗∗*^*P* < 0.001 value <0.05 in *t*-test and AUC >0.85 in ROC analysis were considered significant. The third round of validation was to analyse the transcriptional expression level of ABC genes of CRC patients on the Oncomine database. The threshold sets were as follows: *P* < 0.05; multiple: 2; genetic rank: top 10%; data type: mRNA. It was considered to have obvious biological significance with the ratio >2 or <0.5.

### 2.4. Prognostic Analysis

To explore the potential value of ABC genes in CRC, survival analysis, descriptive statistics, and univariate and multivariate Cox regression analyses were performed in the TCGA-COADREAD dataset (380 patients with CRC and 51 controls). In the survival analysis, the correlation between gene expression levels and survival status was investigated. A chi-squared test was used to observe the correlation between gene expression level and CRC patients' age, gender, clinical stage, tumor site, and living status. In univariate Cox regression analysis, we further explored the relationship between age, sex, clinical stage, tumor site, gene expression level, and CRC survival status. Parameters that were statistically significant in the univariate Cox regression analysis were involved in the multivariate Cox regression analysis. Finally, multivariate Cox regression analysis was used to identify whether gene expression level was an independent prognostic factor in CRC patients. *P* < 0.05 was considered statistically significant in survival analysis, descriptive statistics, and univariate and multivariate Cox regression analyses.

### 2.5. Functional Analysis

#### 2.5.1. PPI Network Construction and Functional Analysis

The STRING (https://string-db.org/) database is used to integrate all known and predicted connections between proteins, including physical interactions and functional associations. We performed PPI (protein-protein interaction) analysis for genes in the STRING database. The basic parameters were set as follows: organism: *Homo sapiens*; minimum required interaction score: medium confidence 0.4; 1st shell: no more than 50 interactors; and the rest of the parameters were default. The interaction relationship for related genes was finally obtained.

To explore the potential pathways and biological processes of the related genes, we used the annotation visualization and integrated discovery database (DAVID 6.8; https://david.ncifcrf.gov/) for GO pathway analysis of interacting genes obtained from STRING database. Cytoscape is one of the most successful tools for network biology analysis and visualization, and we used Cytoscape 3.8.0 (https://cytoscape.org/) tool to construct the PPI network of related genes. In addition, we presented the results of the GO analysis in bubble charts drawn by https://www.bioinformatics.com.cn—a free online platform for analysing and visualizing the data.

#### 2.5.2. GSEA

GSEA was performed using GSEA v4.1.0 software, which was a knowledge-based software for interpreting whole-genome expression profiles and was available for free at https://www.gsea-msigdb.org. The parameters were set as follows: gene sets database: c2.cp.kegg.v7.4.symbols.gmt; number of permutations: 100; collapse: no_collapse; and permutation type: gene_set.

#### 2.5.3. WGCNA and GO Pathway Analysis

WGCNA was conducted using the Sangerbox tools, a free online platform for data analysis (https://www.sangerbox.com/tool). The parameters were set as follows: network type: unsigned; screening genes: no; outlier sample: filter; soft threshold: 3; minimum module size: 60; sensitivity: 2; and module merge threshold: 0.25. The identified modular genes were analysed by the GO pathway in the DAVID database.

### 2.6. Immuno-Infiltration Analysis

The TIMER database (https://cistrome.shinyapps.io/timer/) can be used to systematically analyse the immune infiltrates of different cancer types. Here, we analysed the relationship between the expression level of the correlated genes and the abundance of immune infiltrates in colorectal cancer, including B cells, CD4+ T cells, CD8+ T cells, neutrophils, macrophages, and dendritic cells.

### 2.7. Statistical Analysis

ROC analysis and ROC curves, *t*-test, survival analysis, and survival curves were calculated and plotted by GraphPad prism 8.0. A chi-squared test and univariate and multivariate Cox regression analyses were performed in SPSS 20.0 software. In addition, the datasets were all grouped according to median gene expression level, and *P* < 0.05 were considered statistically significant.

## 3. Results

### 3.1. Identification and Screening

A PubMed search was conducted on June 20, 2021, and we found a total of 27 genes that had not been studied with CRC (Table 1). *t*-test and ROC analysis were performed for the 27 genes in GSE44076 and GSE44861 datasets. When *P* < 0.05 for *t*-test and AUC > 0.5 for ROC analysis were regarded as the limiting conditions, when *t*-test *P* < 0.05 and ROC analysis AUC > 0.5 were the constraints, only seven ABC genes—*ABCA5*, *ABCA8*, *ABCA10*, *ABCC1*, *ABCC13*, *ABCF2*, and *ABCF3*—could enter the validation stages (Table S1).

### 3.2. First Round of Validation

The seven genes screened in the previous round were validated in the GSE9348 dataset. The result showed that there were 6 genes with *P* < 0.05 and AUC > 0.85, namely *ABCA5*, *ABCA8*, *ABCA10*, *ABCC1*, *ABCC13*, and *ABCF2*. Among them, *ABCA5*, *ABCA8*, *ABCC1*, and *ABCC13* in the *t*-test were with *P* < 0.0001, indicating that the difference between the CRC group and the control group was highly significant (Table S2).

### 3.3. Second Round of Validation

**Figure 2:**
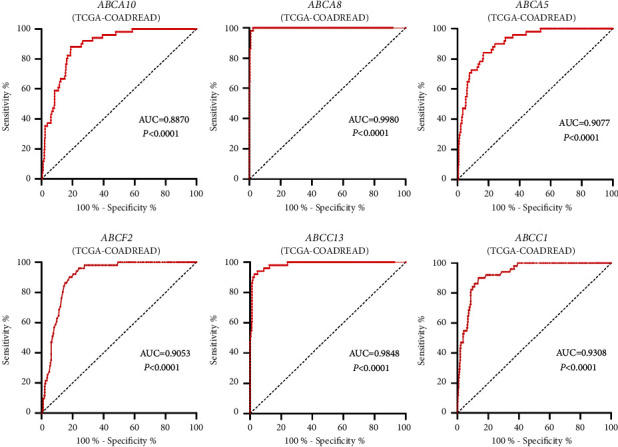
ROC analysis of the expression data for diagnostic assessment of 6 genes according to the TCGA database (AUC statistics are used to evaluate the capacity to discriminate CRC samples from normal controls with specificity and sensitivity).

The second round of validation for these 6 genes was performed in the TCGA-COADREAD dataset. The result showed that they could go to the next round of validation with *P* < 0.05 and AUC >0.85 (Table 2), namely *ABCA5*, *ABCA8*, *ABCA10*, *ABCC13*, *ABCC1*, and *ABCF2*. Among them, five genes in the ROC analysis were with AUC > 0.90, indicating that their sensitivity and specificity are relatively high (Figure 2).

### 3.4. Third Round of Validation

The transcriptional expression level of these six genes was validated in the Oncomine database. The results of *ABCA5*, *ABCA8*, and *ABCC1* were shown in Figure 2S. The figure showed that they were differentially expressed in 20 different types of tumors. *ABCA5* and *ABCA8* were lowly expressed in cancer tissues, and *ABCC1* was highly expressed in colorectal cancer tissues. There were 12 datasets for *ABCA5*, 17 datasets for *ABCA8*, and 7 datasets for *ABCC1* (Tables 3S–S5). No dataset was found about *ABCA10*, *ABCC13*, and *ABCF2*. These datasets could prove that the transcriptional expression levels of *ABCA5*, *ABCA8*, and *ABCC1* are significantly different between the CRC group and the control group, and these three genes would enter the next round of verification.

### 3.5. Prognostic Analysis

**Figure 3:**
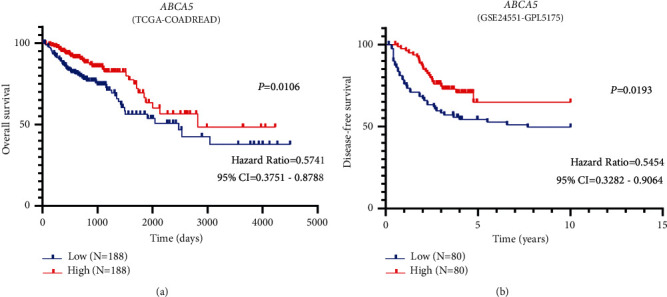
Kaplan–Meier survival curve of *ABCA5* mRNA expression in CRC patients. (a) Survival analysis in TCGA-COADREAD dataset. (b) Survival analysis in GSE24551-GPL5175 dataset.

The survival analysis of *ABCA5*, *ABCA8*, and *ABCC1* genes was performed in the TCGA colon and rectal cancer (TCGA-COADREAD) and GSE24551-GPL5175 datasets. It was found that only *ABCA5* had prognostic significance. In colorectal cancer patients, patients with high expression of *ABCA5* had a better prognosis than those with low expression of *ABCA5* (Figure 3). A chi-squared test was conducted on *ABCA5* to determine whether the expression of *ABCA5* was related to age, gender, and other clinical indicators. The results showed a significant correlation between *ABCA5* gene expression level and the survival status of CRC patients (Table 3). To find out the relationship between patient survival status and clinical characteristics, we performed a univariate Cox regression analysis, including age, gender, clinical stage, T stage, M stage, N stage, tumor tissue site, and transcript expression level of *ABCA5* gene. The result indicated that age, clinical stage, M stage, and *ABCA5* expression level were significantly related to the prognosis (Table 4).

Multivariate Cox regression analysis was performed on age, clinical stage, M stage, and *ABCA5* expression level to determine whether they were independent prognostic factors. The result showed that they were all independent prognostic factors (Table 4). The hazard ratio (HR) for age was 2.866, indicating that the prognosis of CRC patients older than 60 years was worse than that of younger. The hazard ratio for the clinical stage was 2.407, which illustrated that patients with stage III/IV showed a worse prognosis compared to stage I/II. The hazard ratio for the M stage was 2.841, which suggested that patients with distant metastases have a worse prognosis than those without metastases. The hazard ratio for *ABCA5* expression was 0.491, revealing that patients with high expression had a longer survival period than patients with low expression.

### 3.6. Functional Analysis

#### 3.6.1. PPI Network Construction and Functional Analysis for *ABCA5*

**Figure 4:**
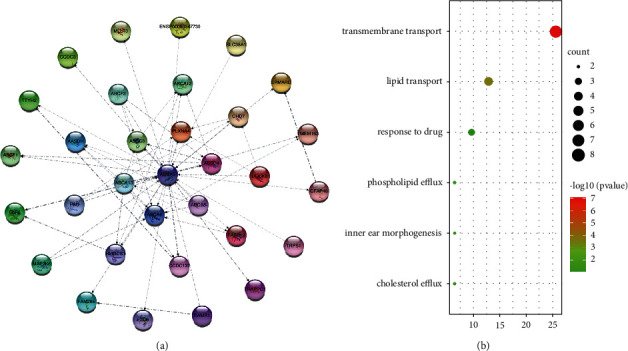
PPI network and functional analysis for *ABCA5*. (a) PPI network from STRING database. (b) Biological process (BP) from GO analysis.

According to the settings, we obtained 31 genes in the STRING database that have direct interaction with *ABCA5* (Figure 4(a)). The thickness of the line represents the strength of the relationship, and the direction of the arrow represents the role of the relationship. The figure shows that there are 10 genes as the source genes of *ABCA5* and 21 genes as the target genes of *ABCA5*. Among them, *ELP6* and *CCDC137*, as the target genes of *ABCA5*, are more closely related to *ABCA5* (Figure 4(a)).

To further understand the biological processes in which *ABCA5* may be involved, we performed GO enrichment analysis on these 31 genes. The result of the biological process (BP) indicated that *ABCA5* is involved in lipid transport and cholesterol efflux, etc (Figure 4(b), Table S6).

#### 3.6.2. GSEA for *ABCA5*

**Figure 5:**
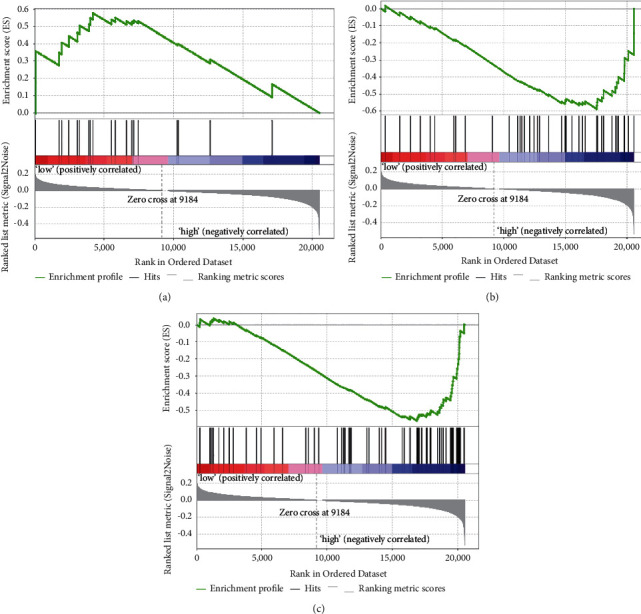
GSEA for ABCA5. (a) Fatty acid synthesis pathway. (b) ABC transport pathway. (c) Drug metabolism pathway.

To understand how *ABCA5* plays a role in the pathway, we did GSEA with TCGA-COADREAD as the background dataset. A total of 176 pathways were enriched, 53 pathways with *P* < 0.05 and FDR< 0.25, including 33 pathways associated with *ABCA5* low expression activation and 20 pathways associated with *ABCA5* high expression activation. We focused on three pathways for analysis: unsaturated fatty acid synthesis pathway, ABC transport pathway, and drug metabolism pathway. In the unsaturated fatty acid synthesis pathway, the peak was in the left low expression group, indicating that the fatty acid synthesis pathway was activated when *ABCA5* was expressed at a low level (normalized enrichment score, NES = 1.89) (Figure 5(a)). In the ABC transport pathway, the peak was in the high expression group on the right, indicating that it was activated when *ABCA5* was expressed at a high level (NES = −1.72) (Figure 5(b)). In the drug metabolism pathway, the peak was in the high expression group on the right, indicating that it was activated when *ABCA5* was expressed at a high level (NES = −1.70) (Figure 5(c)).

#### 3.6.3. WGCNA and GO Analysis for ABCA5

**Figure 6:**
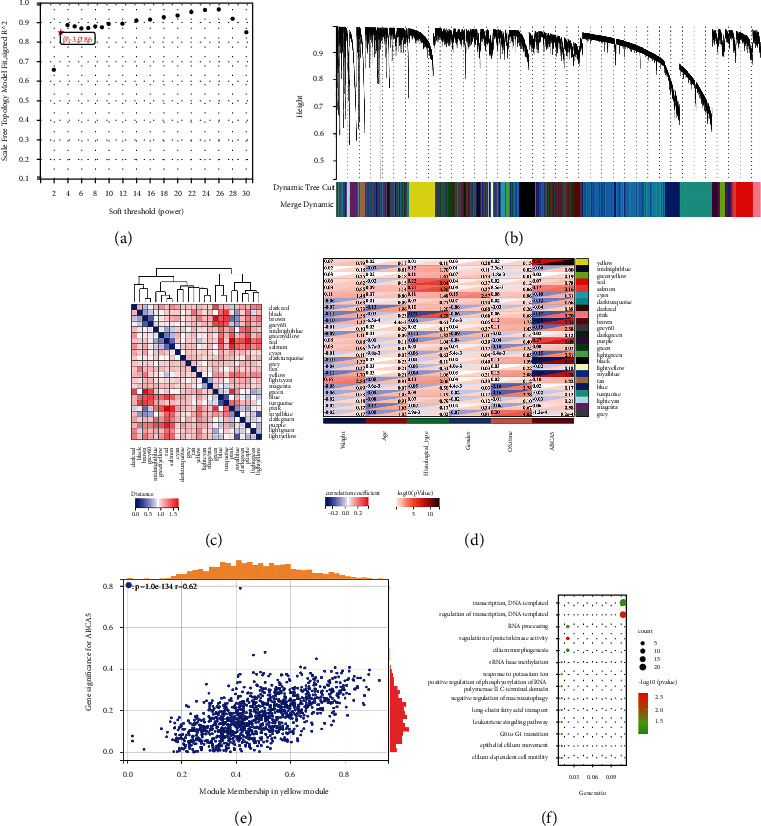
Identification of modules associated with the *ABCA5* expression in the TCGA-COADREAD dataset. (a) Determination of the optimal soft threshold. (b) The cluster dendrogram of co-expression network modules was ordered by a hierarchical clustering of genes based on the 1-TOM matrix. Each module was assigned to different colors. (c) Heat map of module feature vector clustering. (d) Module-trait relationships. Each row corresponds to a color module and column corresponds to a clinical trait. Each cell contains the corresponding correlation and *P* value. (e) Scatterplot of GS vs. MM correlation for yellow module (the corresponding correlation and *P* value). (f) GO analysis for the hub genes of the yellow module.

To discover more potential pathways, we performed WGCNA on *ABCA5*. Gene modules associated with *ABCA5* expression in CRC were identified, and then key modules were selected for GO analysis. Specifically, the network topology under different soft threshold powers was first analysed so that the WGCNA had relatively balanced scale independence and average connectivity. A *β* = 3 power was chosen as a soft threshold in order to ensure that we developed a scale-free network (Figure 6(a)). The average linkage method and Pearson correlation analysis were used to cluster the sample of 383 CRC patients (Figure 6(b)). Next, 24 different gene modules were identified after setting the sensitivity to 2 and combining the modules with less than 25% difference (Figures 6(c) and 6(d)). Finally, a yellow module was identified based on the highest correlation of the module with *ABCA5* expression (Cor = 0.36) (Figures 6(d) and 6(e)).

Subsequently, we extracted 274 hub genes from the yellow module by setting the MM (module membership) threshold to 0.60 and the GS (gene significance) threshold to 0.1. GO enrichment analysis of these genes revealed that they were enriched to 14 biological process (BP) pathways, including transcriptional regulation and transport of fatty acids, leukotriene signalling and macrophage regulation, among other pathways (Figure 6(f)).

### 3.7. Immune Infiltration Analysis for *ABCA5*

**Figure 7:**
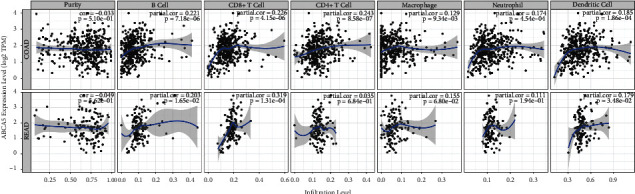
Immune infiltration analysis for *ABCA5*.

The results of immune infiltration analysis showed the correlation between *ABCA5* expression and immune infiltration of colorectal cancer. A positive association was found between the expression level of *ABCA5* and the infiltration levels of B cells, CD8+ T cells, CD4+ T cells, macrophages, neutrophils, and dendritic cells in colon cancer tissues (*P* < 0.05). In rectal cancer, the expression level of *ABCA5* was positively correlated with the infiltration level of B cells and CD8+ T cells, while it was not significantly different from the infiltration level of CD4+ T cells, macrophages, and neutrophils (Figure 7).

In addition, we have done a pan-cancer analysis of *ABCA5* with immune cells. It can be seen from the figure that *ABCA5* is involved in the immune response to a variety of cancers, including BLCA (bladder cancer), COAD (colon adenocarcinoma), BRCA (breast cancer), KICH (kidney chromophobe), KIRC (kidney renal clear cell carcinoma), THCA (thyroid carcinoma), THYM (thymoma), and UCEC (uterine corpus endometrial carcinoma), with strong relevance to immune cells, including KICH, TGCT (tenosynovial giant cell tumor), and THYM (Figure S3).

## 4. Discussion

Many studies have demonstrated that ABC family genes have an important position in the development of diseases [14, 15], cholesterol metabolism [5, 16], and drug resistance [17]. Forty-nine human ABC transporter proteins, about half of which are considered to be involved in the transport of lipids and lipid-related compounds [15]. Jamie et al. proposed that the role of ABC transporter proteins in tumor biology is linked, such as conferring resistance to drug or cancer-related substrates (i.e., phospholipids and cholesterol) [18]. The *ABCA* family consists of 12 isoforms and is the second largest ABC gene family after the *ABCC* family [19]. Evolutionary analysis revealed that the cluster of genes encoding *ABCA5*, *ABCA6*, *ABCA8*, *ABCA9*, and *ABCA10*, referred to as *ABCA5*-like transporter genes, clustered on chromosome 17q24 [19, 20]. Comparative genomic analysis showed that the *ABCA5* gene is relatively evolutionarily conserved, indicating the importance and stability of *ABCA5* in organisms [21, 22]. *ABCA5* gene has been found to act as a biomarker for a variety of diseases. For example, it can be used as a diagnostic marker for prostate cancer and melanoma [23, 24], a key prognostic molecular marker for ovarian cancer, osteosarcoma, and childhood acute myeloid leukemia (AML) [24–26]. After screening, identification, and multiple rounds of validation, *ABCA5* was screened out from 49 ABC transporter protein genes. It was the first time we demonstrated the diagnostic and prognostic value of *ABCA5* in patients with colorectal cancer.

The results of functional analysis from different perspectives suggested that *ABCA5* may be involved in the synthesis of unsaturated fatty acids, cholesterol efflux, and drug metabolism. In recent years, the *ABCA5* gene is reported to be involved in the development and treatment of many diseases, mainly in the aspects of drug resistance and cholesterol transport. *ABCA5* is engaged in resistance processes in the treatment of many diseases, including resistance in melanoma [27], resistance to the immunosuppressant tacrolimus [28], resistance to 5-FU in laryngeal squamous cell carcinoma [29], resistance to arabinoside (Ara-C) and erythromycin (Dnr) in acute myeloid leukemia [30], resistance to doxorubicin (Dox) in malignant mesothelioma (MM) [31], and resistance to cisplatin in lung cancer [32]. Diseases associated with *ABCA5* cholesterol transport function include Alzheimer's disease [33], Parkinson's disease [34], ST-segment elevation myocardial infarction [35], atherosclerosis [36, 37], and excessive hair growth [22]. Notably, the overexpression of *ABCA5* is a protective response in these diseases, and upregulation protects cells from the accumulation of intracellular cholesterol and other sterols [22]. The increased expression level of *ABCA5* promotes cholesterol outflow and tends to maintain the balance of cholesterol transport [35, 38].

The accumulation of cholesterol is a well-known feature of cancer [39]. To meet their continuous growth and proliferation needs, cancer cells would increase the uptake of exogenous lipids or upregulate the synthesis of endogenous cholesterol [40]. Lipid droplets are dynamic organelles that store triglycerides (TG) and cholesterol esters (CE) and are highly accumulated in colon cancer cells [41]. There is a consensus that high cholesterol diet and high level of total serum cholesterol increase the risk of colorectal cancer [42–45]. Cholesterol is metabolized to active derivatives, including cholesterol oxidation products (COP), called oxysterols, which have been shown to alter cell proliferation [46, 47]. For example, a high concentration of 27-hydroxycholesterol (27HC), a kind of oxysterol, significantly increases the release of pro-inflammatory interleukins 6 and 8 and exerts tumor-promoting properties in colorectal cancer [48, 49]. The *ABCA5* gene assists in the transport of cellular cholesterol and is involved in the process of lipid metabolism [50]. The expression of *ABCA5* is strongly induced in the presence of cholesterol, which promotes intracellular cholesterol efflux [38, 50], and the effect is evident in macrophages [36, 50, 51]. Macrophages are involved in the formation of tumor microenvironment [52, 53], growth and metastasis [54–57], and apoptosis [58] and play a critical role in tumor immunity [59]. Cholesterol can modulate the expression of inflammatory cytokines, chemokines, and lymphocyte proliferation in macrophages [60]. This indicates that *ABCA5* may have an important effect in the development of colorectal cancer through cholesterol and macrophages. It is consistent with the results of our previous functional analysis and immune infiltration analysis.

Our study showed that *ABCA5* was down-expressed in colorectal cancer patient tissues compared to normal controls. This is consistent with the expression pattern of *ABCA5* in breast cancer [61], suggesting that *ABCA5* plays an oncogene role in colorectal and breast cancers. In addition, in colorectal cancer patients, low expression level of *ABCA5* was associated with shorter overall survival, similar to the expression pattern of *ABCA5* in plasmacytic ovarian cancer [25]. High expression was associated with a better prognosis, suggesting that *ABCA5* overexpression was a protective response in colorectal cancer. This may be due to the fact that *ABCA5* is strongly induced by cholesterol accumulation and begins to express and act as an oncogene. Perhaps it can be understood that the high expression of *ABCA5* is to maintain the balance of cholesterol transport, which would reduce the cholesterol content in cancer cells. Wu et al. suggested that promoting cholesterol efflux inhibits cancer cell growth and affects cancer cell survival [62]. Lipid metabolism is extensively mediated by the LXR (liver X receptor) and PPAR (proliferator-activated receptor) transcription factor families [63]. Ray et al. found that *ABCA5* is a PPAR family regulatory protein with high specificity for the *γ* isoform. The PPAR*γ* agonists, troglitazone and rosiglitazone, resulted in a robust increase of *ABCA5* expression level in macrophages. In addition, they found that the lipid-lowering drugs, fenofibrate and atorvastatin, also increased the mRNA level of *ABCA5* in macrophages [50]. Some studies have found promising results for statins in the prevention and treatment of colorectal cancer [64–66]. Statins increase the expression of *ABCA5* and are associated with longer patient survival [64, 67]. The topics that are worth exploring in the future include what are the mechanisms involved?; and can other drugs that increase the *ABCA5* expression level also prolong the survival of CRC patients?

In addition, 66 potentially active drugs were identified from 119 substances interacting with *ABCA5* in the Comparative Toxicogenomics Database (CTD) (https://ctdbase.org). The expression of *ABCA5* could be upregulated at the mRNA level by 34 of these drugs, whereas 32 drugs could decrease the expression of *ABCA5* (Table S7). Moreover, five *ABCA5* source genes were identified among the seven *ABCA5*-interacting genes: *ABCA13* [68], *ABCA6* [68], *ABCA8A* [68], *ABCA8B* [68], and *PPARG* [69] (Table S8). We hope that these drugs and genes will serve as a reference for future researchers.

## 5. Conclusion

After one round of identification, two rounds of screening, and three rounds of validation, we identified a new diagnostic and prognostic biomarker for colorectal cancer at the mRNA expression level, namely *ABCA5*. Based on the low expression of *ABCA5* in CRC and the correlation with poor prognosis, it may contribute to the diagnosis and provide new ideas for the development of molecularly targeted drugs in CRC.

## Figures and Tables

**Table 1:** ROC analysis and *t*-test of ABC transporter family based on GSE44076 dataset.

No.	Gene	Expression (CRC)	Expression (normal)	CRC vs normal		AUC	*P*
1	**ABCA2** ^**a**^	2.397	2.438	0.983	↓	0.579	0.0351
2	**ABCA3**	2.709	2.788	0.972	↓	0.654	0.0346
3	**ABCA4**	2.425	2.351	1.031	↑	0.653	0.0002
4	**ABCA5**	4.502	5.747	0.783	↓	0.935	<0.0001
5	**ABCA6**	2.442	2.795	0.874	↓	0.910	<0.0001
6	*ABCA7*	3.327	3.261	1.020	↑	0.551	0.1073
7	**ABCA8**	2.621	3.932	0.667	↓	0.997	<0.0001
8	**ABCA9**	2.636	3.071	0.858	↓	0.930	<0.0001
9	**ABCA10**	3.109	3.737	0.832	↓	0.940	<0.0001
10	*ABCA12*	2.451	2.410	1.017	↑	0.542	0.2877
11	**ABCA13**	2.223	2.111	1.053	↑	0.510	0.0464
12	*ABCB6*	Null	Null	Null	—	Null	Null
13	**ABCB7**	8.012	7.775	1.030	↑	0.680	<0.0001
14	*ABCB8*	3.08	3.084	0.999	↓	0.520	0.8720
15	*ABCB9*	2.964	3.008	0.985	↓	0.597	0.0554
16	**ABCB10**	8.276	8.070	1.026	↑	0.680	<0.0001
17	**ABCC6**	3.800	4.065	0.935	↓	0.716	<0.0001
18	**ABCC8**	2.917	2.954	0.987	↓	0.577	0.0490
19	*ABCC9*	3.061	3.127	0.979	↓	0.502	0.3928
20	*ABCC12*	2.231	2.197	1.015	↑	0.586	0.0598
21	**ABCC13**	2.629	3.810	0.690	↓	0.960	<0.0001
22	**ABCD1**	3.940	3.793	1.039	↑	0.599	0.0090
23	**ABCD2**	1.985	2.059	0.964	↓	0.673	<0.0001
24	**ABCF2**	5.292	4.892	1.082	↑	0.806	<0.0001
25	**ABCF3**	4.837	4.715	1.026	↑	0.692	<0.0001
26	*ABCG4*	2.152	2.177	0.989	↓	0.567	0.0789
27	*ABCG8*	2.252	2.278	0.989	↓	0.544	0.3438

**Table 2:** *t*-test and ROC analysis of ABC transporter family members based on the TCGA-COADREAD dataset.

No.	Gene	Expression (CRC)	Expression (normal)	CRC/normal	AUC	*P*
1	*ABCA5*	7.949	9.385	0.846990	0.9077	<0.0001
2	*ABCA8*	3.319	9.625	0.344831	0.9980	<0.0001
3	*ABCA10*	3.118	5.073	0.614626	0.8870	<0.0001
4	*ABCC1*	10.82	9.718	1.113398	0.9308	<0.0001
5	*ABCC13*	2.929	7.046	0.415697	0.9848	<0.0001
6	*ABCF2*	10.68	10.17	1.050147	0.9053	<0.0001

**Table 3:** Chi-squared test of clinical parameters and *ABCA5* mRNA expression in the TCGA-COADREAD cohort.

Parameters	Group	*ABCA5* mRNA expression		*X* ^2^	*P*
		High (*n* = 188)	Low (*n* = 188)		
Age	≤60	73	70	0.102	0.750
	>60	115	118		
Gender	Female	93	76	3.106	0.078
	Male	95	112		
Clinical stage	I/II	97	96	0.949	0.622
	III/IV	84	81		
	Null	7	11		
*T* stage	1∼2	32	35	0.164	0.686
	3∼4	155	152		
	Null	1	1		
*N* stage	0	105	101	0.129	0.720
	1∼3	82	85		
	Null	1	2		
*M* stage	0	25	130	0.000	1.000
	1	125	26		
	Null	38	32		
Tumor site	Colon	143	140	0.129	0.720
	Rectum	45	48		
Living status	Living	157	134	8.041	0.005
	Dead	31	54		

**Table 4:** Univariate and multivariate Cox regression analyses of clinical parameters according to the TCGA-COADREAD dataset.

Parameters	Univariate analysis		*P*	Multivariate analysis		*P*
	HR	95%CI		HR	95%CI	
*Age*						
≤60 vs >60	3.036	1.665–5.536	0.000	2.866	1.595–5.150	0.000

*Gender*						
Female vs male	0.708	0.427–1.173	0.180	—	—	—

*Clinical stage*						
I/II vs III/IV	4.811	1.175–19.707	0.029	2.407	1.342–4.317	0.003

*T stage*						
1∼2 vs 3∼4	1.176	0.448–3.090	0.742	—	—	—

*N stage*						
0 vs 1∼3	0.495	0.138–1.774	0.280	—	—	—

*M stage*						
0 vs 1	2.650	1.399–5.021	0.003	2.841	1.540–5.242	0.001

*Tumor site*						
Colon vs rectum	0.779	0.451–1.347	0.372	—	—	—

*ABCA5 expression*						
High vs low	0.474	0.283–0.792	0.004	0.491	0.294–0.820	0.007

HR, hazard ratio; CI, confidence interval.

## Data Availability

The data used to support the findings of this study are included within the article.

## Supplementary Materials

Supplementary Materials Tables 1S–8S and Figures 1S–3S have been added to Supplementary Materials, which are freely available in Supplementary materials. Table S1: *t*-test and ROC analysis of ABC transporter family members according to the GSE44861 database. Table S2: *t*-test and ROC analysis of ABC transporter family members according to the GSE9348 database. Table S3: *ABCA5* expression analysis according to the Oncomine database. Table S4: *ABCA8* expression analysis according to the Oncomine database. Table S5: *ABCC1* expression analysis according to the Oncomine database. Table S6: Results of GO enrichment analysis in biological process (BP). Table S7: Chemical interactions of *ABCA5* according to CTD database. Table S8: Target genes of *ABCA5* according to CTD database. Figure S1: Distribution of the *ABCA5* gene in different datasets. Figure S2: The mRNA expression of *ABCA5*, *ABCA8*, and *ABCC1* in different types of cancer by Oncomine analysis. (The number in the colored cell represents the number of analyses meeting thresholds. The cell color is determined by the gene rank. The more intense red (overexpression) or blue (underexpression) indicates a more highly significant overexpressed or underexpressed gene.) Figure S3: Pan-cancer analysis of *ABCA5* and immune cells. (Immune checkpoint: The heat map of immune checkpoint-related gene expression. The abscissa represents different immune checkpoint-related gene, and the ordinate represents different tumor tissues. Each box in the figure represents the correlation analysis between the expression of the selected gene and the immune checkpoint in corresponding tumors. ^*∗*^*P* < 0.05, ^*∗∗*^*P* < 0.01, ^*∗∗∗*^*P* < 0.001^*∗∗∗*^*p* < 0.001, asterisks (^*∗*^) stand for significance levels. Different colors represent the changes of correlation coefficients. Immune correlations: The heat map of *ABCA5* immune score and *ABCA5* gene expression in multiple tumor tissues. The abscissa represents different tumor tissues, and the ordinate represents different immune score. Different colors represent the correlation coefficients. Negative values indicate negative correlations and positive values indicate positive correlations; and the deeper the color, the stronger the correlation. ^*∗*^*P* < 0.05, ^*∗∗*^*P* < 0.01, ^*∗∗∗*^*P* < 0.001, asterisks (^*∗*^) stand for significance levels. The statistical difference between two groups was compared through the Wilcoxon test).
