# Supplementary material for: Identification of ABCA5 among ATP-Binding Cassette Transporter Family as a New Biomarker for Colorectal Cancer
Source: J Oncol. 2022 Jun 22;2022:3399311. doi: 10.1155/2022/3399311 (PMC9242773; doi:10.1155/2022/3399311)
Supplement: Supplementary Materials — Tables 1S–8S and Figures 1S–3S have been added to Supplementary Materials, which are freely available in Supplementary materials. Table S1: t-test and ROC analysis of ABC transporter family members according to the GSE44861 database. Table S2: t-test and ROC analysis of ABC transporter family members according to the GSE9348 database. Table S3: ABCA5 expression analysis according to the Oncomine database. Table S4: ABCA8 expression analysis according to the Oncomine database. Table S5: ABCC1 expression analysis according to the Oncomine database. Table S6: Results of GO enrichment analysis in biological process (BP). Table S7: Chemical interactions of ABCA5 according to CTD database. Table S8: Target genes of ABCA5 according to CTD database. Figure S1: Distribution of the ABCA5 gene in different datasets. Figure S2: The mRNA expression of ABCA5, ABCA8, and ABCC1 in different types of cancer by Oncomine analysis. (The number in the colored cell represents the number of analyses meeting thresholds. The cell color is determined by the gene rank. The more intense red (overexpression) or blue (underexpression) indicates a more highly significant overexpressed or underexpressed gene.) Figure S3: Pan-cancer analysis of ABCA5 and immune cells. (Immune checkpoint: The heat map of immune checkpoint-related gene expression. The abscissa represents different immune checkpoint-related gene, and the ordinate represents different tumor tissues. Each box in the figure represents the correlation analysis between the expression of the selected gene and the immune checkpoint in corresponding tumors. ∗P < 0.05, ∗∗P < 0.01, ∗∗∗P < 0.001∗∗∗p < 0.001, asterisks (∗) stand for significance levels. Different colors represent the changes of correlation coefficients. Immune correlations: The heat map of ABCA5 immune score and ABCA5 gene expression in multiple tumor tissues. The abscissa represents different tumor tissues, and the ordinate represents different immune score. Different colors rep [file 3399311.f1.docx]

**Supplementary materials:**

**Table S1. T-test and ROC analysis of ABC transporter family members according to the GSE44861 database.**

| **No.** | **Gene** | **Expression**  **(CRC)** | **Expression**  **(Normal)** | **CRC/ normal** | **AUC** | ***p*** |
| --- | --- | --- | --- | --- | --- | --- |
| 1 | *ABCA5* | 8.9 | 9.7 | 0.91 | 0.76 | <0.0001 |
| 2 | *ABCA8* | 7.4 | 9.6 | 0.77 | 0.77 | <0.0001 |
| 3 | *ABCA10* | - | - | - | - | - |
| 4 | *ABCC1* | 7.7 | 7.1 | 1.09 | 0.81 | <0.0001 |
| 5 | *ABCC13* | - | - | - | - | - |
| 6 | *ABCF2* | 6.8 | 6.7 | 1.01 | 0.64 | 0.0112 |
| 7 | *ABCF3* | 7.7 | 7.9 | 0.98 | 0.65 | 0.0022 |

**Table S2. T-test and ROC analysis of ABC transporter family members according to the GSE9348**

**database.**

| No. | Gene | Expression  (CRC) | Expression  (Normal) | CRC/ Normal | AUC | p |
| --- | --- | --- | --- | --- | --- | --- |
| 1 | ABCA5 | 364.0 | 1215.0 | 0.30 | 0.99 | <0.0001 |
| 2 | ABCA8 | 49.3 | 659.0 | 0.07 | 0.99 | <0.0001 |
| 3 | ABCA10 | - | - | - | - | - |
| 4 | ABCC1 | 1638.0 | 487.2 | 3.36 | 1.00 | <0.0001 |
| 5 | ABCC13 | 51.1 | 278.3 | 0.18 | 1.00 | <0.0001 |
| 6 | ABCF2 | 664.1 | 414.5 | 1.60 | 0.90 | <0.0001 |

**Table S3. *ABCA5* expression analysis according to the Oncomine database.**

| **Gene** | **Dataset** | **Normal**  **(cases)** | **Tumor**  **(cases)** | **Fold change** | **t-test** | ***p*** |
| --- | --- | --- | --- | --- | --- | --- |
| ***ABCA5*** | Skrzypczak Colorectal 2 | Colon (10) | Colon Carcinoma(5) | -3.069 | -16.187 | 3.65×10-10 |
|  | Beroukhim Renal | Colon (10) | Colon Adenoma (5) | -2.487 | -5.665 | 2.31×10-4 |
|  |  |  |  |  |  |  |
|  | TCGA Colorectal | Colon (19)  Rectum(3) | Colon Mucinous Adenocarcinoma (22) | -3.045 | -9.475 | 3.26×10-12 |
|  | TCGA Colorectal | Colon(19)  Rectum(3) | Rectal Mucinous Adenocarcinoma (6) | -3.682 | -9.123 | 2.41×10-6 |
|  | TCGA Colorectal | Colon (19)  Rectum(3) | Colon Adenocarcinoma (101) | -3.434 | -12.948 | 3.37×10-17 |
|  | TCGA Colorectal | Colon (19)  Rectum(3) | Rectal Adenocarcinoma (60) | -3.096 | -11.188 | 9.87×10-16 |
|  | TCGA Colorectal | Colon (19)  Rectum(3) | Cecum Adenocarcinoma (22) | -3.287 | -8.484 | 1.83×10-10 |
|  | TCGA Colorectal | Colon (19)  Rectum (3) | Rectosigmoid Adenocarcinoma (3) | -4.745 | -10.485 | 3.30×10-4 |
|  | Hong Colorectal | Colon (12) | Colorectal Carcinoma (70) | -3.375 | -11.503 | 1.17×10-11 |
|  | Kaiser Colon | Colon (5) | Rectal Adenocarcinoma (8) | -2.127 | -5.301 | 1.28×10-4 |
|  | Kaiser Colon | Colon (5) | Rectosigmoid Adenocarcinoma (10) | -2.03 | -5.578 | 8.07×10-5 |
|  | Kaiser Colon | Colon (5) | Colon Mucinous Adenocarcinoma (13) | -2.104 | -6.465 | 4.54×10-5 |

**Table S4. *ABCA8* expression analysis according to the Oncomine database**

| **Gene** | **Dataset** | **Normal**  **(cases)** | **Tumor**  **(cases)** | **Fold change** | **t-test** | ***p*** |
| --- | --- | --- | --- | --- | --- | --- |
| ***ABCA8*** | TCGA Colorectal | Colon (19)  Rectum (3) | Cecum Adenocarcinoma (22) | -14.099 | -24.859 | 8.89×10-27 |
|  | TCGA Colorectal | Colon (19)  Rectum (3) | Rectal Adenocarcinoma (60) | -14.331 | -27.409 | 2.0×10-36 |
|  | TCGA Colorectal | Colon (19)  Rectum (3) | Colon Mucinous Adenocarcinoma (22) | -16.231 | -20.015 | 1.22×10-20 |
|  | TCGA Colorectal | Colon (19)  Rectum (3) | Colon Adenocarcinoma (101) | -14.271 | -30.144 | 9.92×10-35 |
|  | TCGA Colorectal | Colon (19)  Rectum (3) | Rectal Mucinous Adenocarcinoma (6) | -14.471 | -14,571 | 1.08×10-6 |
|  | TCGA Colorectal | Colon (19)  Rectum (3) | Rectosigmoid Adenocarcinoma (3) | -13.38 | -19.225 | 3.69×10-5 |
|  | Skrzypczak Colorectal | Colorectal Tissue (24) | Colorectal Carcinoma (36) | -16.458 | -15.21 | 1.97×10-21 |
|  |  |  |  |  |  |  |
|  | Skrzypczak Colorectal | Colorectal Tissue (24) | Colorectal Adenocarcinoma (45) | -18.025 | -20.397 | 4.14×10-30 |
|  |  |  |  |  |  |  |
|  | Gaedcke Colorectal | Rectum (65) | Rectal Adenocarcinoma (65) | -6.264 | -23.902 | 5.75×10-45 |
|  | Kaiser Colon | Colon (5) | Rectosigmoid Adenocarcinoma (10) | -10.346 | -13.612 | 6.45×10-9 |
|  | Kaiser Colon | Colon (5) | Cecum Adenocarcinoma (17) | -10.393 | -14.374 | 1.41×10-9 |
|  | Kaiser Colon | Colon (5) | Colon Mucinous Adenocarcinoma (13) | -8.715 | -8.61 | 1.13×10-7 |
|  | Kaiser Colon | Colon (5) | Rectal Mucinous Adenocarcinoma (4) | -9.86 | -10.285 | 6.85×10-5 |
|  | Kaiser Colon | Colon (5) | Colon Adenocarcinoma (41) | -9.442 | -15.467 | 1.56×10-8 |
|  |  |  |  |  |  |  |
|  | Kaiser Colon | Colon (5) | Rectal Adenocarcinoma (8) | -6.274 | -6.673 | 3.50×10-5 |
|  |  |  |  |  |  |  |
|  | Skrzypczak Colorectal 2 | Colon (10) | Colon Carcinoma (5) | -16.97 | -11.764 | 1.24×10-7 |
|  |  |  |  |  |  |  |
|  |  |  |  |  |  |  |
|  | Hong Colorectal | Colon (12) | Colorectal Carcinoma (70) | -24.602 | -12.459 | 3.99×10-11 |

**Table S5. *ABCC1* expression analysis according to the Oncomine database.**

| Gene | Dataset | Normal (cases) | Tumor (cases) | Fold change | t-test | p |
| --- | --- | --- | --- | --- | --- | --- |
| *ABCC1* | Skrzypczak Colorectal 2 | Colon(10) | Colon Carcinoma (5) | 2.02 | 12.46 | 2.06E-08 |
|  | Hong Colorectal | Colon(12) | Colorectal Carcinoma (70) | 3.824 | 19.25 | 3.37E-18 |
|  | Gaedcke Colorectal | Rectum(65) | Rectal Adenocarcinoma (65) | 2.25 | 16.09 | 2.32E-31 |
|  | Skrzypczak Colorectal | Colorectal Tissue (24) | Colorectal Adenocarcinoma (45) | 2.156 | 9.234 | 2.97E-13 |
|  | TCGA Colorectal | Colon(19) Rectum(3) | Cecum Adenocarcinoma (22) | 2.289 | 8.667 | 4.03E-11 |
|  | TCGA Colorectal | Colon(19) Rectum(3) | Colon Adenocarcinoma (101) | 2.055 | 10.401 | 5.86E-14 |
|  | TCGA Colorectal | Colon(19) Rectum(3) | Rectal Adenocarcinoma (60) | 2.037 | 8.936 | 4.62E-13 |

**Table S6. Results of GO enrichment analysis in biological process (BP).**

| **Category** | **Term** | **Count** | **Rate(%)** | **P value** | **Genes** |
| --- | --- | --- | --- | --- | --- |
| GOTERM_BP_DIRECT | GO:0055085~transmembrane transport | 8 | 25.80645 | 8.70E-08 | ABCD4, ABCA5, ABCC8, ABCA6, ABCA8, ABCA12, ABCA13, ABCF1 |
| GOTERM_BP_DIRECT | GO:0006869~lipid transport | 4 | 12.90323 | 2.41E-04 | ABCA5, ABCA6, ABCA8, ABCA12 |
| GOTERM_BP_DIRECT | GO:0033700~phospholipid efflux | 2 | 6.451613 | 0.022285 | ABCA12, ABCA13 |
| GOTERM_BP_DIRECT | GO:0033344~cholesterol efflux | 2 | 6.451613 | 0.039459 | ABCA5, ABCA13 |
| GOTERM_BP_DIRECT | GO:0042472~inner ear morphogenesis | 2 | 6.451613 | 0.08039 | FZD6, CHD7 |
| GOTERM_BP_DIRECT | GO:0042493~response to drug | 3 | 9.677419 | 0.085278 | HMGCS1, ABCC8, MAP2K6 |

**Table S7. Chemical interactions of *ABCA5* according to CTD database.**

| **No.** | **Interacting Chemical** | **Expression** | **References(PubMed ID)** |
| --- | --- | --- | --- |
| 1 | 2,3,7,8-tetrachlorodibenzofuran | ↓ | 32109520 |
| 2 | 3-dinitrobenzene | ↓ | 21983209 |
| 3 | 4-(5-benzo(1,3)dioxol-5-yl-4-pyridin-2-yl-1H-imidazol-2-yl)benzamide | ↓ | 27188386 |
| 4 | abrine | ↑ | 31054353 |
| 5 | acetamide | ↓ | 31881176 |
| 6 | Acetylcysteine | ↓ | 31004932 |
| 7 | Aflatoxin B1 | ↓ | 27153756/22100608 |
| 8 | Aflatoxin B1 | ↑ | 19770486 |
| 9 | Benzo(a)pyrene | ↑ | 30453624/21632980 |
| 10 | bisphenol A | ↓ | 30816183/25181050 |
| 11 | bisphenol A | ↑ | 26063408 |
| 12 | caffeic acid phenethyl ester | ↓ | 20360939 |
| 13 | Carbon Tetrachloride | ↑ | 31919559/31150630 |
| 14 | Chlordecone | ↑ | 29980752 |
| 15 | Chlorpyrifos | ↓ | 21356183 |
| 16 | Choline | ↑ | 20938992 |
| 17 | Clorgyline | ↑ | 19691856 |
| 18 | Cyclosporine | ↓ | 20106945 |
| 19 | Cyclosporine | ↑ | 21632981 |
| 20 | Diethylnitrosamine | ↓ | 20360939 |
| 21 | Dihydrotestosterone | ↓ | 29458080 |
| 22 | Dihydrotestosterone | ↑ | 29581250 |
| 23 | Docetaxel | ↓ | 19944135 |
| 24 | domoic acid | ↑ | 18936300 |
| 25 | dorsomorphin | ↓ | 27188386 |
| 26 | Doxorubicin | ↓ | 29803840 |
| 27 | entinostat | ↓ | 26272509 |
| 28 | entinostat | ↓ | 27188386 |
| 29 | Ethinyl Estradiol | ↑ | 29097150 |
| 30 | fenoxycarb | ↑ | 31927065 |
| 31 | Fenretinide | ↑ | 28973697 |
| 32 | Folic Acid | ↓ | 25629700 |
| 33 | Folic Acid | ↑ | 20938992 |
| 34 | furan | ↑ | 25539665 |
| 35 | Genistein | ↑ | 32186404 |
| 36 | Ionomycin | ↓ | 25613284 |
| 37 | jinfukang | ↓ | 27392435 |
| 38 | (+)-JQ1 compound | ↑ | 26752646 |
| 39 | lipopolysaccharide, E coli O55-B5 | ↑ | 24972896 |
| 40 | masitinib | ↑ | 22747577 |
| 41 | Methionine | ↑ | 20938992 |
| 42 | Methoprene | ↑ | 31927065 |
| 43 | methylmercuric chloride | ↓ | 28001369/26272509/23179753 |
| 44 | Methyl Methanesulfonate | ↓ | 26011545 |
| 45 | NSC 689534 | ↑ | 20971185 |
| 46 | Paclitaxel | ↑ | 20737486 |
| 47 | Panobinostat | ↓ | 27188386 |
| 48 | Panobinostat | ↓ | 26272509 |
| 49 | Pentachlorophenol | ↑ | 27181905 |
| 50 | Phthalic Acids | ↑ | 21061450 |
| 51 | Quercetin | ↓ | 21632981 |
| 52 | systhane | ↑ | 32268158 |
| 53 | Tetrachlorodibenzodioxin | ↑ | 21632981/20106945 |
| 54 | Tetrachloroethylene | ↑ | 28973375 |
| 55 | Tetradecanoylphorbol Acetate | ↓ | 25613284 |
| 56 | Thioacetamide | ↑ | 23411599 |
| 57 | Tretinoin | ↑ | 21934132 |
| 58 | trichostatin A | ↓ | 27188386 |
| 59 | trichostatin A | ↓ | 26272509/24935251 |
| 60 | trichostatin A | ↑ | 24935251 |
| 61 | tris(1,3-dichloro-2-propyl)phosphate | ↑ | 26179874 |
| 62 | Valproic Acid | ↓ | 27188386 |
| 63 | Valproic Acid | ↓ | 28001369/26272509/24383497/23179753 |
| 64 | Valproic Acid | ↑ | 19136453 |
| 65 | Vanadates | ↓ | 22714537 |
| 66 | Vincristine | ↓ | 19944135 |

**Table S8. Target genes of *ABCA5* according to CTD database.**

| **No.** | **Source Gene** | **Target Gene** | **Source Organism** | **Target Organism** | **Interaction Type** | **References (PMID)** |
| --- | --- | --- | --- | --- | --- | --- |
| 1 | ABCA13 | ABCA5 | musculus | musculus | physical | 32325033 |
| 2 | ABCA5 | CYP2E1 | sapiens | sapiens | physical | 21988832 |
| 3 | ABCA5 | NR4A1 | sapiens | sapiens | physical | 21988832 |
| 4 | ABCA6 | ABCA5 | musculus | musculus | physical | 32325033 |
| 5 | ABCA8A | ABCA5 | musculus | musculus | physical | 32325033 |
| 6 | ABCA8B | ABCA5 | musculus | musculus | physical | 32325033 |
| 7 | PPARG | ABCA5 | musculus | sapiens | physical | 27197753 |


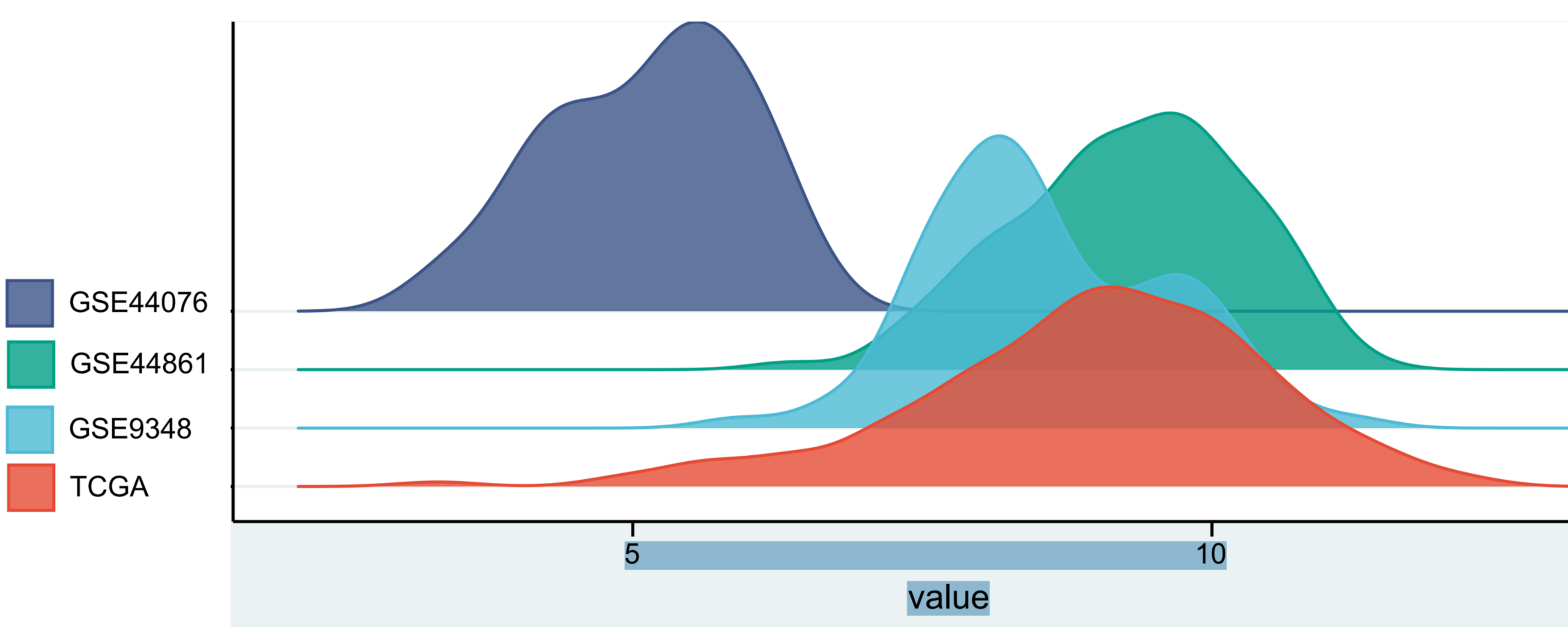


**Figure S1. Distribution of the *ABCA5* gene in different datasets.**


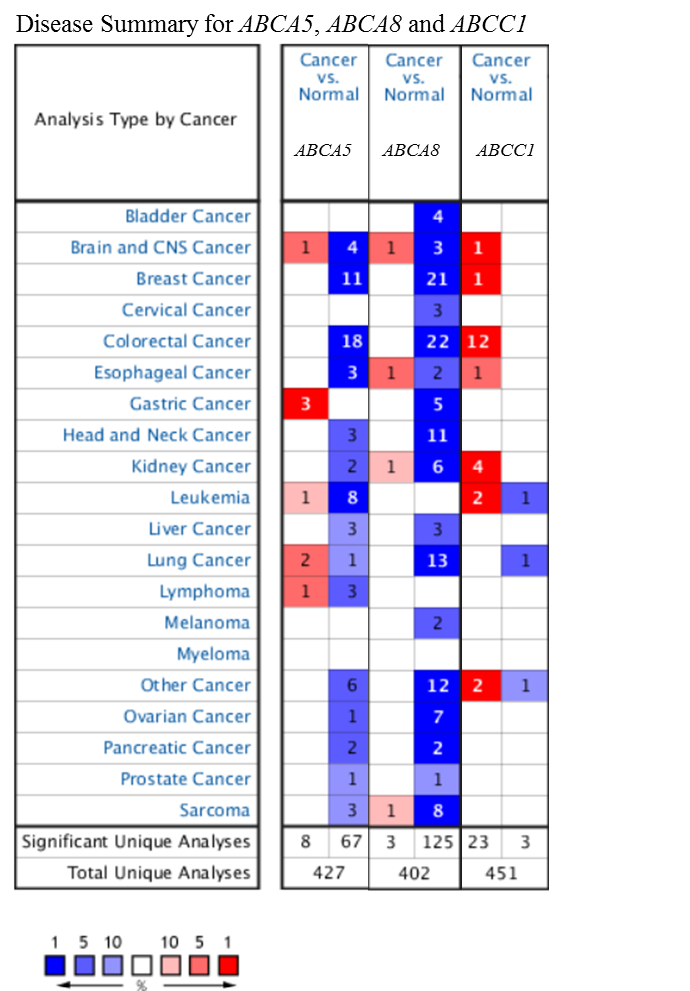


**Figure S2. The mRNA expression of *ABCA5*, *ABCA8* and *ABCC1* in different types of cancer by Oncomine analysis. (The number in the colored cell represents the number of analyses meeting thresholds. Cell color is determined by the gene rank. The more intense red (over-expression) or blue (under-expression) indicates a more highly significant over-expressed or under-expressed gene.)**


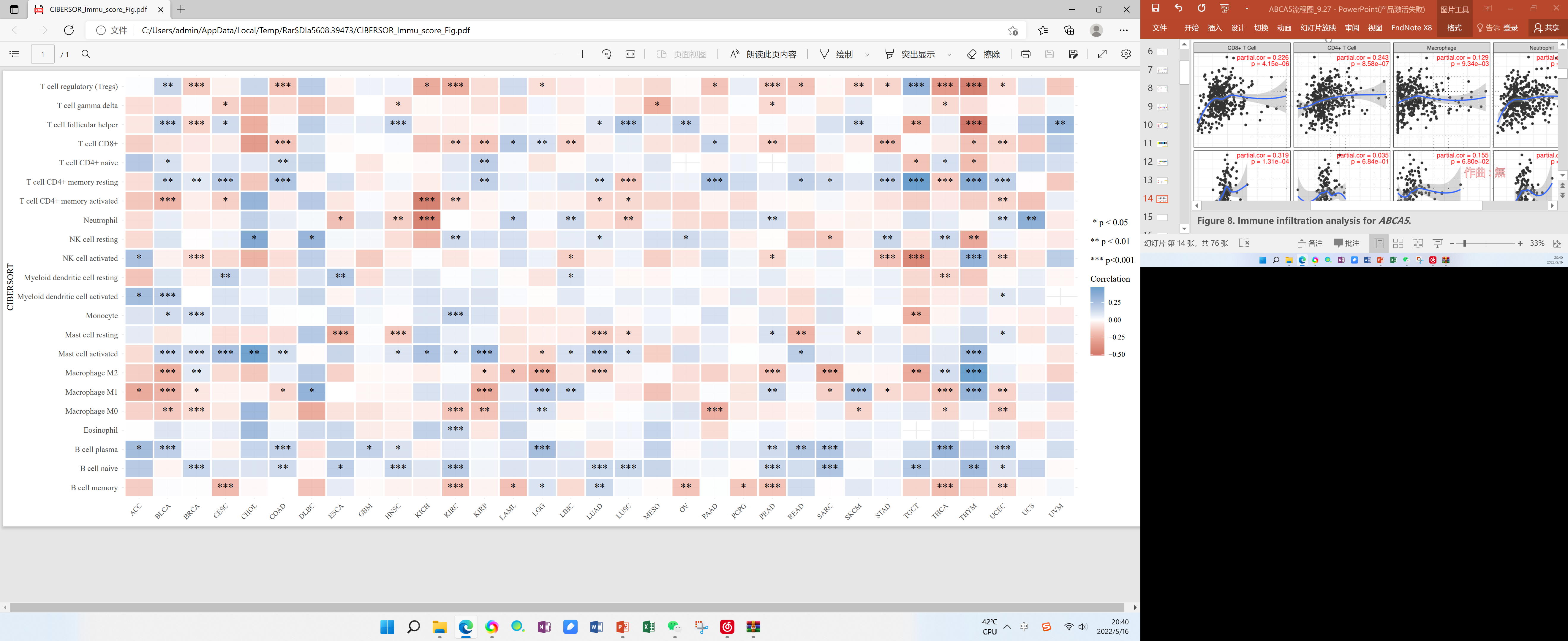


**Figure S3. Pan-cancer analysis of *ABCA5* and immune cells. (Immune Checkpoint: The heatmap of immune-checkpoint-related gene expression. The abscissa represents different immune-checkpoint-related gene, and the ordinate represents different tumor tissues. Each box in the figure represents the correlation analysis between the expression of the selected gene and the immune checkpoint in corresponding tumors. *p < 0.05, **p < 0.01, ***p < 0.001, asterisks (*) stand for significance levels. Different colors represent the changes of correlation coefficients. Immune Correlations: The heatmap of *ABCA5* immune score and *ABCA5* gene expression in multiple tumor tissues. The abscissa represents different tumor tissues, and the ordinate represents different immune score. Different colors represent the correlation coefficients. Negative values indicate negative correlations and positive values indicate positive correlations, the deeper color the stronger correlation. *p < 0.05, **p < 0.01, ***p < 0.001, asterisks (*) stand for significance levels. The statistical difference of two groups was compared through the Wilcox test.)**
